# Auditory Sensory Gating: Effects of Noise

**DOI:** 10.3390/biology13060443

**Published:** 2024-06-18

**Authors:** Fan-Yin Cheng, Julia Campbell, Chang Liu

**Affiliations:** Department of Speech, Language, and Hearing Sciences, University of Texas at Austin, Austin, TX 78712, USA; fanyin.cheng@austin.utexas.edu (F.-Y.C.); changliu@austin.utexas.edu (C.L.)

**Keywords:** central inhibition, informational masking, energetic masking, cortical auditory evoked potentials, gating

## Abstract

**Simple Summary:**

Many individuals report difficulty perceiving speech in background noise. Research has shown that one factor in this type of processing is the effect of noise on the brain’s response to speech. Different types of noise affect the strength of the neural encoding of speech to varying degrees, with greater informational masking causing greater degradation. Previous research has focused on the effects of attention in noise, which tends to enhance the neural encoding of the speech signal via inhibitory mechanisms. However, there is a pre-attentive stage of inhibition, sensory gating, which has been linked to speech perception in noise. The impact of noise on the neural response during this phase remains unclear. This preliminary study investigated the gating response, recorded via high-density EEG in fifteen normal-hearing adults, in quiet and noise conditions with low and high informational masking. Scalp maps were generated to observe brain activation in each condition. Significant gating occurred in quiet, while noise conditions resulted in a significantly decreased gating response. The gating function was significantly degraded in noise with less informational masking content, coinciding with a reduced activation of inhibitory gating networks. These findings illustrate the adverse effect of noise on pre-attentive inhibition related to speech perception.

**Abstract:**

Cortical auditory evoked potentials (CAEPs) indicate that noise degrades auditory neural encoding, causing decreased peak amplitude and increased peak latency. Different types of noise affect CAEP responses, with greater informational masking causing additional degradation. In noisy conditions, attention can improve target signals’ neural encoding, reflected by an increased CAEP amplitude, which may be facilitated through various inhibitory mechanisms at both pre-attentive and attentive levels. While previous research has mainly focused on inhibition effects during attentive auditory processing in noise, the impact of noise on the neural response during the pre-attentive phase remains unclear. Therefore, this preliminary study aimed to assess the auditory gating response, reflective of the sensory inhibitory stage, to repeated vowel pairs presented in background noise. CAEPs were recorded via high-density EEG in fifteen normal-hearing adults in quiet and noise conditions with low and high informational masking. The difference between the average CAEP peak amplitude evoked by each vowel in the pair was compared across conditions. Scalp maps were generated to observe general cortical inhibitory networks in each condition. Significant gating occurred in quiet, while noise conditions resulted in a significantly decreased gating response. The gating function was significantly degraded in noise with less informational masking content, coinciding with a reduced activation of inhibitory gating networks. These findings illustrate the adverse effect of noise on pre-attentive inhibition related to speech perception.

## 1. Introduction

Various electrophysiological studies using cortical auditory evoked potentials (CAEPs) have shown that background noise significantly affects the pre-attentive, bottom-up neural encoding of sound at the level of the cortex, typically resulting in decreased peak component (P50, N1, P2) amplitude and increased latency [1,2,3,4,5,6,7,8,9,10,11,12,13,14,15]. However, these effects are heavily dependent on extrinsic factors such as noise type [4,10,12,15]. Noise type may be categorized by whether there are high levels of energetic masking (i.e., peripheral masking, due to spectral interactions between the signal and noise) or informational masking (i.e., increased stimulus uncertainty or similar semantic and contextual properties between the signal and noise) [16,17,18,19,20,21]. For example, in stationary noise such as speech-shaped noise (SSN), there is little change in spectrotemporal properties over time, and no meaningful (i.e., speech-related) content to interfere with the target signal, resulting in mainly energetic masking [10,22]. Because cortical neurons respond to changes in stimuli rather than ongoing input, CAEP responses in this type of noise are more robust (i.e., present with higher amplitude and a clear waveform morphology) [3,12] and dependent upon the signal-to-noise ratio (SNR) [3,5,6,10,14]. Indeed, decrements in CAEP amplitude and increased latency evoked during continuous noise with a decreasing SNR correspond with the behavioral perception of speech in noise dominated by energetic masking [5,22]. In speech-modulated noise (e.g., spectrally and temporally matched with speech), both energetic and informational masking have been shown to be present due to stimulus uncertainty and the similarity in temporal fluctuations between the signal and noise [10,17]. CAEP components tend to be more affected by this condition than stationary noise, illustrating a degraded neural encoding of sound as the informational masking increases [10,15]. In multi-talker babble noise, informational masking has been demonstrated to be significantly present, as there is a larger degree of stimulus uncertainty and similarity between the signal and distractor, specifically for speech stimuli [4,17]. The CAEP morphology is significantly deteriorated (i.e., decreased amplitude and indistinct waveform morphology) in this condition, particularly the N1 and P2 components, which are sensitive to stimulus characteristics and higher-order pre-cognitive processing [2,4,10,12,15,23,24]. These findings are again consistent with poorer speech perception performance in noise with increased levels of informational masking [22,25]. In other words, both electrophysiological and perceptual data support the finding that it is more difficult to encode and subsequently perceive speech in the presence of high levels of informational masking.

While extrinsic factors clearly influence the neural encoding of sound in noise, intrinsic factors, such as attention, also modulate the CAEP response [2,4,11,15,26]. Generally, in background noise with a greater degree of energetic masking (e.g., stationary noise), attention to the stimulus increases the amplitude of the N1 and P2 peak components [4,11,15], although it should be noted that the analysis of the P50 component is not always included [11,15]. Similar findings are described in background noise with increased informational masking (e.g., multi-talker babble) [2,4,15]. The findings of increased neural amplitude during attention might reflect various inhibitory mechanisms that are implemented to help segregate the signal from the background noise. One possible scenario is that the neural response to ongoing noise is actively suppressed by top-down modulation from cognitive centers in the frontal and pre-frontal cortex, aiding in the successful encoding and processing of the signal [27,28,29,30,31,32].

However, it is important to note that there is a pre-attentive inhibitory stage, termed sensory inhibition, that is responsible for the filtering of information before it reaches higher-order, or conscious, attention centers. These centers may include, but are not limited to, the dorsolateral region of the pre-frontal cortex [33,34] as well as the orbitofrontal cortex and the anterior cingulate [35]. During this automatic process, repetitive non-novel input is suppressed by top-down modulation via the frontal and pre-frontal cortex without requiring the listener’s active or conscious attention [36,37,38,39]. Sensory inhibition, or gating, is measured using CAEPs elicited by identical pairs of sounds such as tones, clicks, and speech. While a click stimulus has been traditionally used, recent studies have reported the implementation of speech sound pairs [40,41,42,43,44,45], with several using vowel pairs [40,41,42,43]. These studies have reported robust gating responses using such stimuli, with the results having a greater real-world application than non-speech sounds. The sensory gating response is quantified by comparing P50, N1, and P2 peak amplitude values between the response evoked by the second sound (S2) and the response evoked by the first sound (S1) in the pair [36]. When typical sensory gating occurs, the response to the S2 stimulus is suppressed, and the amplitude difference between CAEP S1 and S2 (i.e., CAEP S1-S2) should increase, while the amplitude ratio (CAEP S2/S1) decreases [36,46,47]. The P50 gating component is traditionally viewed as a biomarker of sensory inhibition, more so than the N1 and P2 components [36,47]. Nonetheless, these components may also reflect pre-attentive inhibition via amplitude suppression [48,49].

A significant relationship has been described between the sensory stage of pre-attentive inhibition and mild SPiN deficits in adults with normal hearing [27,44]. Those with a greater CAEP S1-S2 amplitude difference require a lower SNR to successfully perceive SPiN [27,44]. However, the gating paradigm in these studies was conducted in quiet, leaving the direct effects of noise on the neural encoding of sound during this specific inhibitory stage unknown. Therefore, the first aim of this preliminary study was to investigate the effects of noise on the sensory inhibitory function using a passive auditory gating paradigm in quiet, multi-talker babble-modulated noise, and multi-talker babble conditions. Based on previous research reporting degraded neural encoding in noise via CAEPs, we hypothesized that the CAEP gating response in noise would be decreased, reflecting a negative impact of noise on automatic sensory inhibitory mechanisms. Indeed, the introduction of background noise degraded the overall gating response. The second aim of this study was to determine whether background noise with lower informational masking content (i.e., multi-talker babble-modulated noise) or higher informational masking content (i.e., multi-talker babble) would present with differential effects on the gating function. As higher informational content tends to result in greater deterioration of the CAEP response, we hypothesized that the presence of noise with higher informational masking content would result in a significantly degraded gating response when compared to the other listening environments. This hypothesis was not supported, as the condition with lower informational masking resulted in a greater deterioration of the gating function.

## 2. Materials and Methods

### 2.1. Participants

Fifteen participants (M = 26 years, SD = 2.27 years) were enrolled in this study, which took place at the Central Sensory Processes Laboratory at the University of Texas at Austin.

All participants provided written informed consent to participate in the study, and the University of Texas at Austin Institutional Review Board approved all procedures (protocol #2015-09-0154). The participants presented with auditory function within clinical normal limits as assessed by otoscopy, immittance, and conventional audiometry conducted bilaterally across the clinical frequency range of 150–8000 Hz via 3M E-A-R TONE GOLD 3A insert earphones. Thresholds of ≤20 dB HL were required at each octave within the clinical frequency range to be defined as normal hearing for study inclusion [50]. Participants were excluded if they had a history of hearing, speech, or language disorders or neurological diagnoses. Participants were asked about their smoking status, as nicotine use affects gating measures [39], and all reported a “nonsmoker” status. No participants reported having difficulties with processing speech in noise. Two participants were excluded due to an absence of the P50-N1-P2 complex, leaving a total of 13 subjects for data analysis (mean age = 26 years old, SD = 2.31 years).

### 2.2. Auditory Gating Paradigm

For the EEG recording session, participants were fitted with a 128-channel electrode net (Electrical Geodesics, Inc., Eugene, OR, USA) and seated in a reclining chair located in an electromagnetically shielded sound booth. The online reference electrode was designated at Cz. Ocular artifacts were recorded through designated eye electrodes for offline rejection. The sampling rate for the EEG recording was 1000 Hz, with a band-pass filter set at 0.1–200 Hz [27,51,52]. The gating paradigm consisted of 300 vowel /I/ pairs pre-recorded by native English male speakers and presented using E-Prime (Psychology Software Tools). The vowel stimulus was modified with a scale function designed to calculate the root mean square (RMS) sound pressure level (SPL) of long-term speech signals and was calibrated at 80 dB SPL in MATLAB (MathWorks). The vowel pairs were presented at 80 dB SPL via two PSB Alpha B speakers placed in a sound field at a +/−45° azimuth. The interstimulus interval was 600 ms, and the inter-trial interval was 1.5 s [53]. The noise conditions (quiet, babble-modulated, and multi-talker babble-modulated noise) were presented in a random order, with a short break between each condition per participant request. Participants watched a muted movie with subtitles during EEG recordings to reduce the effects of attention [54].

A vowel stimulus was implemented in this study to remain consistent with the use of speech stimuli used in previous CAEP-in-noise research. The short vowel /I/ was chosen because it includes relatively more phonological cues in 50 ms than the long vowel /i/ when listeners categorize speech [55], resulting in high intelligibility scores (e.g., >98%; [56]). To our knowledge, four studies have used a vowel stimulus in a gating paradigm, which was /a/ (F0 = 140 Hz) [40,41,42,43]. The vowel /I/ in the present study had a similar F0 value to that stimulus, at 124.98 Hz. This stimulus also had a shorter vowel duration (50 ms [55] versus 170 ms [40,41,42,43]), with the purpose of increasing the number of testing trials (300 trials vs. 120 trials [43]) for an increased peak SNR and decreased testing time.

The speech signal was presented in quiet and at 5 dB SNR [5] in two noise conditions: four-talker babble-modulated noise and four-talker babble. Babble-modulated noise comprised the background noise condition with the lowest level of informational masking, and it was generated using a Gaussian noise passed through a spectral filter that was matched with the long-term speech spectrum of the four-talker babble. The result was long-term speech-shaped noise (LTSSN). Next, the LTSSN was multiplied by the temporal envelope of the four-talker babble generated in this study [57]. Four-talker babble was designated as the background noise condition with the highest level of informational masking, and it has been used in several studies to assess behavior and neural encoding in the presence of informational masking content [4,10,58,59]. It was generated by mixing speech recordings that were originally recorded from two female and two male English native speakers using speech materials from the Child Encyclopedia [60]. Before being mixed, the speech recordings were calibrated to the same root mean square level.

It should be noted that, although the babble-modulated noise primarily provided energetic masking, it may have contained some informational masking due to the stimulus (masker) uncertainty and the listener’s familiarity with the speech-like temporal fluctuation. However, as the babble-modulated and multi-talker babble-modulated noise were matched and differed only according to speech content (i.e., no speech content was present in the babble-modulated noise), increased informational masking in the multi-talker babble may be mainly attributed to speech content. Indeed, the increase in informational masking present in the multi-talker babble condition has been quantified in several behavioral studies using the same noise generation approach described in the present study [57,61,62,63]. These studies evaluated the amount of informational masking induced by the speech content of six-talker babble on vowel identification for English and Mandarin Chinese native speakers. Overall, listeners underperformed in multi-talker babble significantly, more so than in babble-modulated noise, across multiple SNR levels. For example, young normal-hearing listeners with Mandarin Chinese as their native language achieved a vowel identification score in the multi-talker babble condition (SNR of −8 dB) of 30% below the average score demonstrated in babble-modulated noise [57]. In another study of English vowel identification in noise, Mi et al. (2021) [63] found that the informational masking of six-talker English babble (calculated by subtracting the vowel identification scores in the babble condition from the scores in babble-modulated noise) ranged from 5% to 15% for English native speakers across SNRs of −9 to 3 dB. Thus, given the findings of previous physiological and behavioral studies, the vowel /I/ was selected as the target signal to be embedded in babble-modulated noise and four-talker babble in this study, with increased informational masking present in the four-talker babble condition.

### 2.3. Electroencephalography Analysis

EEG data were high-pass filtered offline at 1 Hz and segmented to a −100 ms pre-stimulus and 600 ms post-stimulus epoch. Data were exported from Net Station into EEGLAB [64]. The event segments were baseline-corrected to the pre-stimulus interval. Channels with a high level of artifacts for over 50% of the recording were removed and re-referenced using the average reference, excluding ocular channels [27,51,52,65]. No more than 28 channels were removed, based on the finding that dipole location is not improved with more than 100 electrodes [66]. Thus, for scalp map purposes, we retained the necessary number of electrodes to view accurate locations of cortical activation, especially following interpolation (see below). Data were down-sampled to 250 Hz, which decreased the independent component analysis (ICA) processing time and is part of standard procedure in such analyses [27,65]. A total of 300 segments comprised each CAEP S1 and S2 average per listening condition. Next, the Infomax algorithm for ICA was performed on individual concatenated CAEP S1 and S2 segments for the purpose of artifact rejection. ICA provides separation of independent sources that are linearly mixed across sensors [67], and it allows for artifact removal without rejecting entire trial segments, maximizing the signal-to-noise ratio. Components that replicated the CAEP morphology, demonstrated peak electrical activity over the frontal, central, and temporal regions, and displayed consistent inter-trial coherence over the 300 segments were retained. Inter-trial coherence is a measure provided by EEGLAB which illustrates the strength of a peak response over concatenated trials or segments, and it allows for the observation of the consistency of a peak response [68]. A robust cortical response will present with consecutive peak activity over all segments during the peak time period of interest [68], while myogenic activity will tend to be ‘smeared’ over time. Components with a poor morphology, atypical scalp maps (e.g., positive polarity over ocular regions in the case of ocular activity), and absent inter-trial coherence were rejected. A total of 64 components were analyzed for each participant in each listening condition. On average, 3–8 components were retained for each individual CAEP S1 and S2, numbers which agree with previous ICA results used to identify components for source localization of the gating response in our lab [23,27,65,69,70]. Once the components making up the gating response were identified, missing channels were interpolated via a spherical interpolation algorithm [23,27,51,52,65,69]. The concatenated event segments in each individual CAEP S1 and S2 data set were then averaged.

Keeping in line with the previous research [23,27,51,52,65,69], an individual frontal region of interest (ROI) was created from an average of thirteen electrodes (3, 4, 5, 9, or Fp2, 10, 11, or Fz, 12, 15, 16, 18, 19, 22, or Fp1, 23) (note that Electrical Geodesics electrode locations do not follow the 10-20 system). The CAEP peaks in response to S1 and S2 were identified visually by the first author and marked at the highest peak point or mid-peak for a broad peak [23,27,51,52,65,69]. Peaks were identified as P50 being the first positive peak, N1 the first negative peak, and P2 the second positive peak following N1. Approximate latency ranges were as follows: P50, 45–90 ms; N1, 80–200 ms; and P2, 110–250 ms. Please see Table 1 for mean latency values for P50, N1, and P2 components across noise conditions. A 30 Hz low-pass filter was applied for figure purposes only. The peak component amplitude-gating difference (e.g., P50 amplitude S1–P50 amplitude S2) was calculated for P50, N1, and P2 peaks for each participant to quantify the inhibitory function. The amplitude-gating difference index was chosen to quantify sensory inhibition based on studies that found this measure to be more sensitive to differences in inhibitory function than the more commonly used amplitude-gating ratio [27,71].

### 2.4. Data Analysis

A two-way repeated measures analysis of variance (ANOVA) was conducted to evaluate the effect of noise conditions (quiet, multi-talker babble-modulated noise, and babble-modulated noise) on CAEP within-condition-S1 peak amplitude (P50, N1, P2) and S2 peak amplitude. To further explore the effects of noise conditions and stimuli (S1 vs. S2), pairwise comparisons with a Bonferroni adjustment were employed. Additionally, a one-way repeated measures ANOVA was performed to examine the effect of noise conditions (quiet, multi-talker babble-modulated noise, and babble-modulated noise) on CAEP P50, N1, and P2 peak amplitude-gating difference values. Shapiro–Wilk tests confirmed the normality assumption for amplitude within noise conditions and stimuli, as well as amplitude-gating difference values across conditions. Extreme outliers violating this assumption were removed from the analysis. All statistical analyses were conducted using R (version 4.2.1). A *p* value of 0.05 or less was considered statistically significant.

## 3. Results

### 3.1. P50 Gating

The results of the two-way ANOVA with repeated measures showed that there was no significant interaction effect between noise conditions and stimuli (F(1.31, 14.37) = 4.06, *p* = 0.05, η^2^ = 0.07). Additionally, there was no significant main effect for stimuli (F(1, 11) = 4.31, *p* = 0.06, η^2^ = 0.06). However, there was a significant main effect for noise conditions (F(2, 22) = 5.88, *p* < 0.05, η^2^ = 0.18), indicating that noise conditions had an individual influence on amplitude measurements.

**Figure 1:**
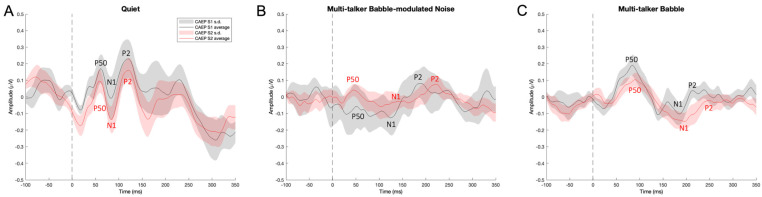
Grand average CAEP P50. N1, P2 gating responses. The CAEP gating responses include the average of 300 CAEP S1 and S2 trials across participants (n = 13) in each listening condition at the frontal ROI. The black line indicates the cortical auditory evoked potential (CAEP) response to stimulus 1 (S1), and the red line indicates the CAEP response to stimulus 2 (S2). The shaded areas indicate the standard deviation of the average CAEP response to S1 (in gray) and S2 (in red). The y-axis represents amplitude (μV) as a function of the x-axis, time (ms). (**A**) CAEP gating in quiet. (**B**) CAEP gating in multi-talker babble-modulated noise. (**C**) CAEP gating in multi-talker babble.

**Figure 2:**
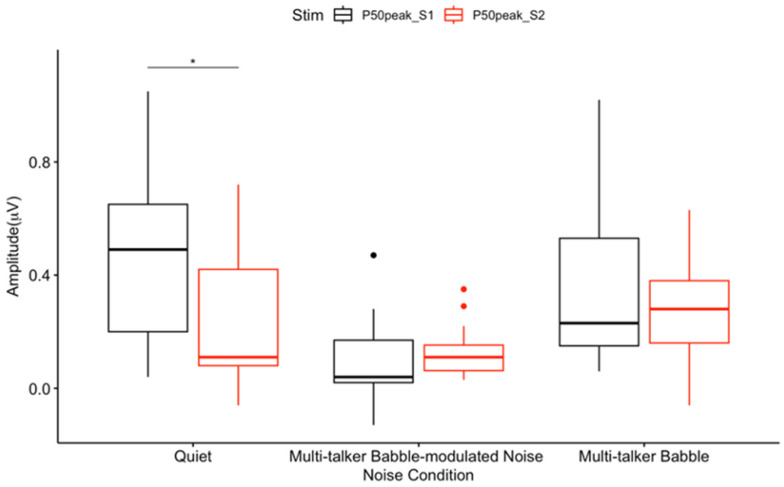
Mean CAEP P50 peak amplitudes. Peak amplitude includes the average of 300 CAEP S1 and S2 trials across participants (n = 13) in each listening condition at the frontal ROI. The box and whisker plot illustrates P50 S2 amplitude suppression. The y-axis represents amplitude (μV), and the x-axis represents the three noise conditions. The horizontal bar indicates the median, the vertical bar indicates the range, and the dot indicates data points out of range. The box represents the mean plus one standard deviation. * *p* < 0.05.

Given the borderline significance (*p* = 0.05) of the interaction effects between noise conditions and stimuli, pairwise comparisons were conducted to further explore the effects of noise conditions and stimuli. The results showed a trend of decreasing P50 amplitudes from quiet to multi-talker babble to multi-talker babble-modulated noise for S1. Among all comparisons, a significant CAEP P50 S2 amplitude suppression was observed for participants in the quiet condition (*p* < 0.05) (Figure 1A and Figure 2), but not in multi-talker babble-modulated noise (*p* = 0.61), or multi-talker babble (*p* = 0.24) conditions. These results are in line with the typical P50 S2 amplitude suppression observed in quiet [23,27,51,52,65,69], and demonstrate that the paradigm was successful in eliciting a baseline gating response. There was an overall visual trend for P50 S2 amplitude to be decreased in multi-talker babble and multi-talker babble-modulated noise (Figure 1B,C and Figure 2).

**Figure 3:**
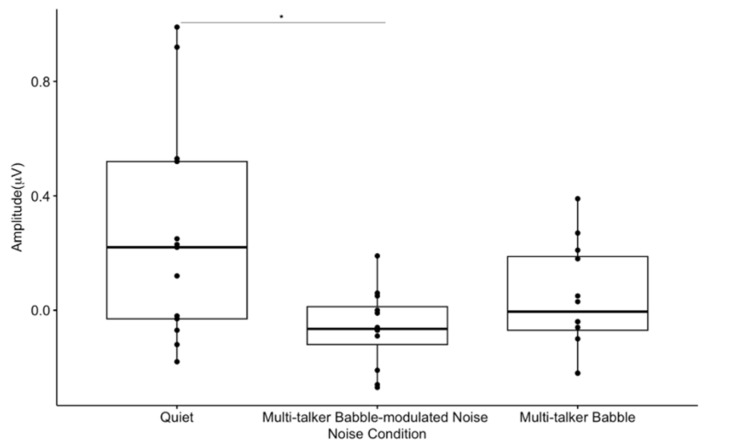
Mean P50 amplitude-gating differences. The amplitude-gating differences include the difference between the average of 300 CAEP S1 and S2 trials across participants in each listening condition at the frontal ROI. The horizontal bar indicates the median, the vertical bar indicates the range, and the dot indicates data points. The box represents the mean plus one standard deviation. * *p* < 0.05.

P50 amplitude-gating differences significantly differed between noise conditions (F(1.34, 14.76) = 5.08, *p* < 0.05, η^2^ = 0.22) (Figure 3). Pairwise comparisons showed that there was a greater amplitude-gating difference recorded in quiet than in multi-talker babble-modulated noise (*p* < 0.05), while the amplitude-gating difference in quiet versus multi-talker babble (*p* = 0.22) presented with a non-significant weak trend prior to a correction for multiple comparisons. There was no significant difference between P50 amplitude-gating difference values in the two noise conditions (*p* = 0.36), suggesting that both low and high levels of informational masking affect pre-attentive levels of inhibition to a similar degree. This result does not support our hypothesis that noise with a higher amount of informational masking would result in a decreased gating function.

### 3.2. N1 Gating

**Figure 4:**
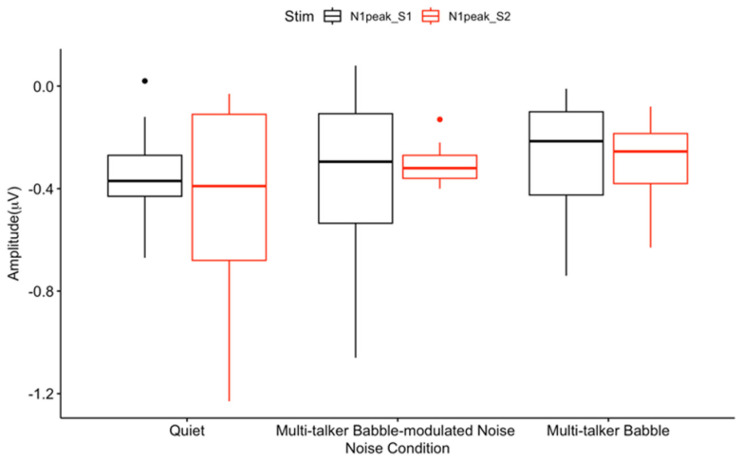
Mean CAEP N1 peak amplitudes. The peak amplitudes include the average of 300 CAEP S1 and S2 trials across participants in each listening condition at the frontal ROI. The box and whisker plot illustrates the trend for N1 S1 and S2 amplitude differences. The y-axis illustrates amplitude (μV), and the three listening conditions are presented on the x-axis. The horizontal bar indicates the median, the vertical bar indicates the range, and the dot indicates data points out of range. The box represents the mean plus one standard deviation. * *p* < 0.05.

The two-way ANOVA with repeated measures indicated no significant interaction effect between noise conditions and stimulus (F(2, 12) = 0.34, *p* = 0.72, η^2^ = 0.01). Also, there was no significant main effect for noise conditions (F(2, 12) = 0.17, *p* = 0.85, η^2^ = 0.02) and stimuli (F(1, 6) = 0.21, *p* = 0.67, η^2^ = 0.002) (Figure 1 and Figure 4), which disagrees with recent results of a gating paradigm performed in informational masking [72]. However, we have observed a lack of N1 and S2 amplitude suppression in previous gating studies performed in quiet in our lab [27,51,53,65] showing that this a consistent finding in typical adults, at least for the frontal ROI.

**Figure 5:**
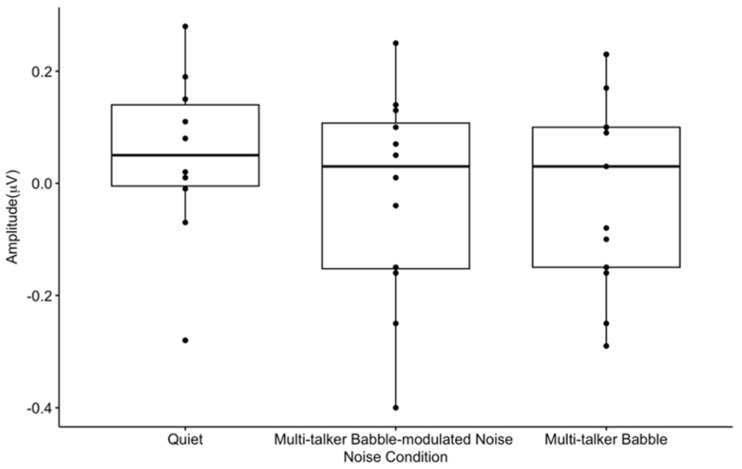
Mean N1 amplitude-gating differences. The amplitude-gating differences include the difference between the average of 300 CAEP S1 and S2 trials across participants in each listening condition at the frontal ROI. The horizontal bar indicates the median, the vertical bar indicates the range, and the dot indicates data points. The box represents the mean plus one standard deviation. * *p* < 0.05.

No statistically significant differences in N1 amplitude-gating differences were observed across listening conditions (F(2, 16) = 0.94, *p* = 0.41, η^2^ = 0.07) (Figure 5), a result inconsistent with findings of significantly decreased gating in an informational masking condition versus quiet [72].

### 3.3. P2 Gating

**Figure 6:**
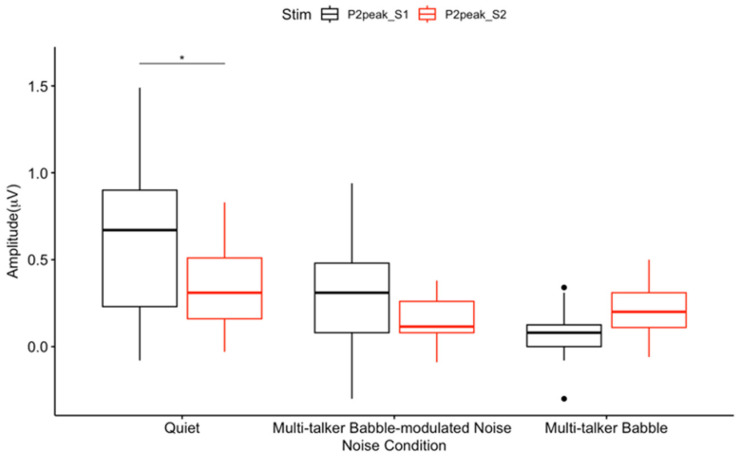
Mean CAEP P2 peak amplitudes. The peak amplitude and latencies include the average of 300 CAEP S1 and S2 trials across participants in each listening condition at the frontal ROI. The box and whisker plot illustrates the trend for P2 S2 amplitude suppression. The y-axis illustrates amplitude (μV), and the three listening conditions are presented on the x-axis. The horizontal bar indicates the median, the vertical bar indicates the range, and the dot indicates data points out of range. The box represents the mean plus one standard deviation. * *p* < 0.05.

The two-way ANOVA with repeated measures indicated no significant interaction effect between noise conditions and stimuli (F(2, 16) = 2.13, *p* < 0.05, η^2^ = 0.05). Pairwise comparisons were conducted to further explore the effects of noise conditions and stimuli. The results showed that significant P2 gating occurred in the quiet condition (*p* < 0.05), which has been reported in other studies [48,49,73]. However, there was no statistically significant difference in P2 S1 and S2 amplitude gating in either noise condition (multi-talker babble-modulated noise: *p* = 0.3, multi-talker babble: *p* = 0.16) (Figure 1B,C and Figure 6).

**Figure 7:**
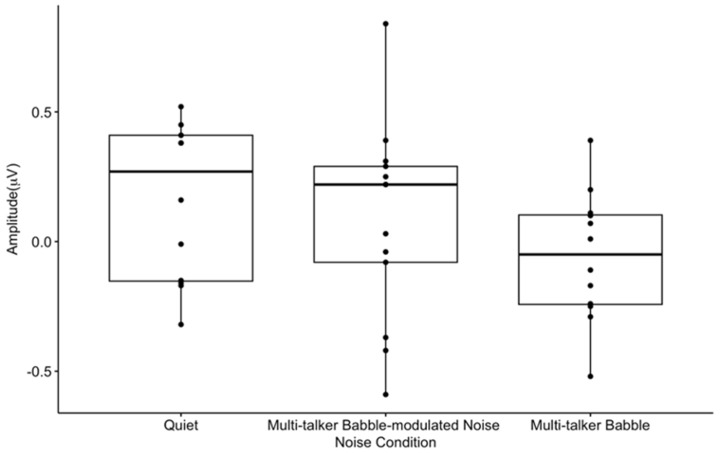
Mean P2 amplitude-gating differences. The amplitude-gating differences include the difference between the average of 300 CAEP S1 and S2 trials across participants in each listening condition at the frontal ROI. The horizontal bar indicates the median, the vertical bar indicates the range, and the dot indicates data points. The box represents the mean plus one standard deviation. * *p* < 0.05.

There were no statistically significant differences in P2 amplitude-gating differences across listening conditions (F(2, 20) = 2.1, *p* = 0.15, η^2^ = 0.15) (Figure 7). This lack of significance does not support the hypothesis that the degree of informational masking in noise may differentially affect pre-attentive inhibition.

### 3.4. P50 Gating Scalp Maps

**Figure 8:**
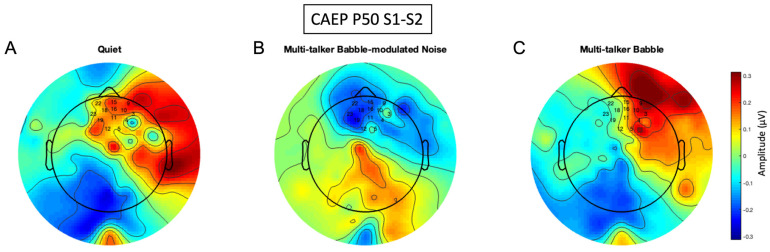
P50 amplitude difference scalp maps. The scalp maps were generated using the frontal region of interest (ROI) and are comprised of the difference of CAEP S1 and S2 averages of 300 trials across all participants (n = 13) in each listening condition. (**A**) CAEP gating in quiet. (**B**) CAEP gating in multi-talker babble-modulated noise. (**C**) CAEP gating in multi-talker babble. Electrode numbers denote the frontal ROI.

Topographic scalp maps generated during the CAEP P50 S1-S2 time frame (Figure 8) illustrate that the gating function was generated in slightly right-lateralized frontal, pre-frontal, and temporal cortical networks in quiet. The gating response in the babble-modulated noise condition shows activity as blue, due to the negative polarity of the response (Figure 1B). The networks underlying this response are also in the right fronto-temporal region but should be interpreted with caution, as the gating waveform morphology was poor in this condition and may not have been a true response. In the multi-talker babble condition, similar gating networks are observed as in the quiet condition, but at a lower intensity. These data agree with the pattern of P50 amplitude difference indices across noise conditions (Figure 3). Activation in the temporal, prefrontal, and frontal regions is also observed in research demonstrating that these inhibitory areas are activated simultaneously with the auditory cortex [39,65] and may modulate auditory cortical processing. Overall, these amplitude difference scalp maps indicate a largely negative effect of noise on P50 cortical networks.

### 3.5. N1 Gating Scalp Maps

**Figure 9:**
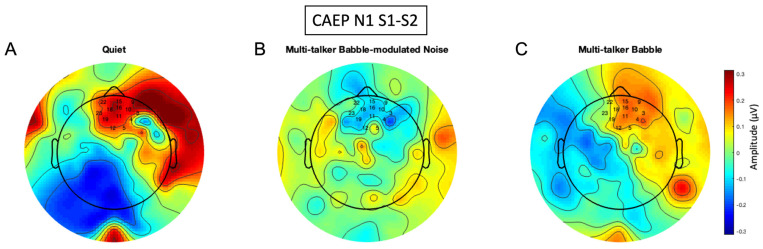
N1 amplitude difference scalp maps. The scalp maps were generated using the frontal region of interest (ROI) and are comprised of the difference of CAEP S1 and S2 averages of 300 trials across all participants (n = 13) in each listening condition. (**A**) CAEP gating in quiet. (**B**) CAEP gating in multi-talker babble-modulated noise. (**C**) CAEP gating in multi-talker babble. Electrode numbers denote the frontal ROI.

Typically, N1 amplitude-gating differences should result in fronto-central activation denoted by negative polarity [72]. However, due to the positive amplitude-gating difference values for the N1 component in quiet and multi-talker babble (Figure 1A,C), activity underlying the N1 gating response is illustrated by positive polarity (red regions in Figure 9A,C). This result is due to the N1 S2 peak having increased in amplitude, versus being suppressed in comparison to the N1 S1 (Figure 4). In quiet, the N1 gating network is located in the frontal, pre-frontal, and temporal regions. While these sources agree with previous studies [27,65,74], this activation represents a stronger S2 response that is atypical, or reflective of decreased inhibition [36]. For the multi-talker babble-modulated noise condition, the gating response is illustrated in blue due to the negative amplitude difference value (Figure 5). Again, this component was degraded in the presence of increased energetic masking content, illustrated by the lack of inhibitory network activation present in the scalp map. In the multi-talker babble condition, gating networks in the frontal, pre-frontal, and temporal regions were observed, but this activity arose from an increased S2 response (Figure 4). This finding differs from a recent study which showed N1 topographic scalp maps to highlight central gating networks in the presence of noise with informational masking content [72]. Such a discrepancy may be due to stimulus differences (speech versus white noise bursts) and/or the SNR of the stimulus to masking (5 dB versus 10 dB), which degraded the listening condition for the present study.

### 3.6. P2 Gating Scalp Maps

**Figure 10:**
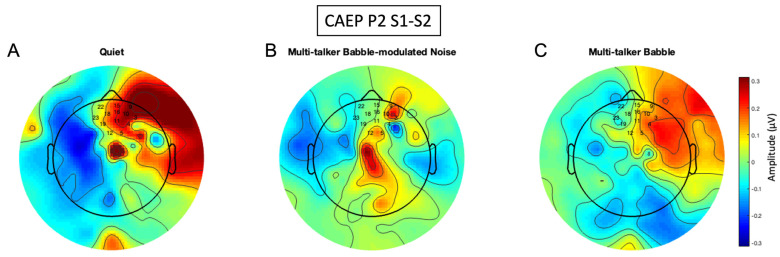
P2 amplitude difference scalp maps. The scalp maps were generated using the frontal region of interest (ROI) and are comprised of the difference of CAEP S1 and S2 averages of 300 trials across all participants (n = 13) in each listening condition. (**A**) CAEP gating in quiet. (**B**) CAEP gating in multi-talker babble-modulated noise. (**C**) CAEP gating in multi-talker babble. Electrode numbers denote the frontal ROI.

Similar to the P50, the P2 gating networks were found in the frontal, pre-frontal, and temporal regions for quiet and multi-talker babble conditions (Figure 10A,C), in agreement with previous studies performed in quiet [27,65]. The right hemispheric lateralization is consistent with previous gating research in typical populations in quiet, and may be related to the processing of prosodic information of speech stimuli [27,65,75]. In contrast, the P2 gating response in multi-talker babble-modulated noise is diminished (Figure 10B), with minimal activation observed in the central and right fronto-temporal networks.

## 4. Discussion

The first aim of this preliminary study was to observe the effects of noise on pre-attentive inhibitory mechanisms using a sensory gating paradigm. The second aim was to determine if there were differential effects of noise with low (i.e., multi-talker babble-modulated noise) and high informational masking content (i.e., multi-talker babble) on the auditory gating response. CAEPs were recorded via high-density EEG and evoked by vowel pairs in quiet, multi-talker babble-modulated noise, and multi-talker babble. CAEP S1 and S2 P50, N1, and P2 peak amplitudes and latencies were compared within condition for each listening environment in order to assess successful inhibitory function. Amplitude-gating difference indices were compared across conditions for each component to determine whether noise with a low or high informational masking content resulted in distinct changes in inhibitory gating. Finally, difference wave scalp maps were generated to qualitatively observe the general activation of inhibitory cortical networks. Two main findings are noted: First, a significant inhibition was found to occur only in the quiet condition, via the P50 and P2 gating response. Overall, the presence of noise greatly degraded the morphology of the gating response in each noise condition by decreasing the CAEP peak amplitude, resulting in less robust peaks in comparison to the quiet condition. There was a visual trend for the most degraded CAEP morphology to occur during background noise with less informational masking content, with nearly absent salient peak responses. The scalp map topography of the gating components illustrated the subsequently decreased or absent activation of typical inhibitory networks. These preliminary results support our hypothesis that the pre-attentive inhibitory function during background noise would be decreased. Second, the P50 gating significantly differed between quiet and noise with a lower degree of informational masking, indicating that sensory inhibition was compromised in this particular noise condition. However, there was no significant difference in gating function between the low or high informational masking noise backgrounds, suggestive of comparable noise effects on inhibitory gating regardless of the noise content. This result does not support our hypothesis that increased informational masking would have a greater negative impact on pre-attentive inhibition. Thus, noise overall seems to interfere with the gating function at a sensory or pre-attentive inhibitory stage, but further research is needed to determine whether specific noise content may present with distinct effects on the gating response.

### 4.1. General Noise Effects on Auditory Sensory Gating

Within-condition CAEP S1 and S2 amplitude comparisons showed that there was a significant P50 and P2 S2 amplitude suppression in quiet (Figure 1A, Figure 2 and Figure 6). This finding agrees with the gating literature, particularly for the P50 gating component, which is described as a biomarker of sensory inhibition useful in examining atypical inhibition in clinical populations [36,47]. P2 gating in quiet has also been shown to occur in typical populations, although it is not as heavily studied as P50 [46,47,49,71,76]. These results are notable, as the gating paradigm implemented in this study consisted of a 50 ms /I/, a short vowel stimulus that has not been previously evaluated in gating research. Speech stimuli are not traditionally used to assess sensory inhibition, with tones and clicks constituting the majority of described gating protocols [27,38,39,46,47,71,76]. Four studies have successfully utilized a 170 ms /a/, which has been reported to evoke a successful P50 S2 amplitude suppression [40,41,42,43]. Therefore, speech stimuli gating paradigms may be applicable in future research evaluating sensory inhibition related to speech processing [40,41,42,43,44,45].

When background noise with either a lower (multi-talker babble-modulated noise) or higher informational masking content (multi-talker babble) was introduced to the gating paradigm, the CAEP morphology underlying the gating response was markedly degraded (Figure 1B,C). Similar morphological effects have been described in several studies examining the cortical encoding of sound in noise, likely due to interactions between the signal and the spectrotemporal properties of the noise [10,11,15]. This acoustic interference may make it difficult for cortical neurons to fire in response to the onset of the target stimulus, as shared neuronal groups are firing to the signal and noise fluctuations simultaneously [3,15]. Indeed, significant CAEP S2 amplitude suppression was found to be absent for the P50 and P2 components. Therefore, it appears that noise had the overall effect of reducing the automatic, pre-attentive inhibitory function, as the cortical encoding of sound was degraded. This may be one reason why higher-order or conscious inhibitory processes related to attention are necessary to improve the neural response in background noise [2,4,11,15]. In other words, sensory inhibitory mechanisms may not be sufficient in suppressing noise interference effects on the neural encoding of sound, requiring attention to be directed to the signal for inhibition to be effective. One way to test this theory would be to evaluate sensory gating in noise with and without attention. Another consideration is that this result is specific to the stimuli factors of this study. For instance, Major and colleagues [72] showed decreased, but present, gating in multi-talker babble at an SNR of 10 dB. Future research should then systematically examine the effects of stimulus, noise type, SNR, and attention on the sensory gating response. In any case, the preliminary findings of an absent CAEP gating agrees with the hypothesis of decreased sensory inhibition in noise.

This hypothesis is further qualitatively supported by the scalp map topography of CAEP gating components in quiet and in noise (Figure 8, Figure 9 and Figure 10). P50 inhibitory gating activity in quiet is depicted in the pre-frontal, frontal, and temporal regions, sensory inhibitory generators identified in source localization studies [27,38,65]. It is of interest that responses were overall lateralized to the right hemisphere, a finding which warrants further exploration. P2 gating is shown in similar regions in quiet, also supported by previous findings [27,65]. The N1 gating activity is atypical due to a larger S2 versus S1 amplitude, but the inhibitory regions agree with source localization findings of generators in the frontal cortex [27,65]. In general, noise resulted in decreased activation of inhibitory networks across all components, with a possible absence of a network response in background noise with lower informational masking (multi-talker modulated babble). Thus, sensory inhibition appears to be decreased in background noise, at least for the conditions of the gating paradigm implemented in this study.

### 4.2. Absent Differential Effects of Noise on Auditory Sensory Gating

A significant gating difference between listening conditions was found only for the P50 gating component. P50 gating was reduced in background noise with lower informational masking content when compared to quiet, and it approached a significant decrease with higher informational masking prior to a correction for multiple comparisons (Figure 3). A recent study reported noise with informational masking to decrease N1 gating when compared to a quiet listening condition; however, P50 gating in quiet was not established [72]. Similarly, the N1 and P2 components have been consistently shown to reflect noise effects, especially because the N1 component is affected by signal audibility and acoustic properties [2,5,15,24]. Therefore, it is of interest that this study found the P50 gating response, considered to be a biomarker of sensory inhibition, to be sensitive to noise effects in comparison to the N1 and P2 components. This may indicate that inhibition at an earlier sensory inhibitory processing stage (i.e., pre-attentive) is more susceptible to noise interference. In addition, the gating response did not significantly differ between noise with lower versus higher informational masking content (multi-talker modulated babble vs. multi-talker babble), which may imply that sensory inhibitory mechanisms are not dependent upon the type of masking content comprising the noise. Rather, if sufficient interference is present to degrade the gating response, sensory inhibition may be absent. This finding conflicts with research reporting poor CAEP encoding and sound perception as the informational masking content of noise increases [2,4,10,12,15,17,22]. For example, we would expect the cortical response to be stronger in modulated noise, which contains amplitude fluctuations and no speech content, allowing neurons to fire distinctly in response to the target signal during points of low energy [4,12]. It is possible that the lack of significant difference in the gating response between the two noise backgrounds is related to the babble-modulated noise used for the low informational masking condition. This noise was chosen because it allows for a direct comparison with multi-talker babble, with the main difference being an absence of linguistic information. CAEP gating may be differentially sensitive to masking content when noise with a greater amount of energetic masking content is utilized, such as steady-state noise. Despite the lack of statistical significance between the gating function in the two noise conditions, there was a visual trend for gating to be more reduced across components when less informational masking content was present, both in CAEP waveform amplitude and topographic scalp maps. Again, this non-significant trend does not agree with worse neural encoding and behavioral performance in noise with a higher degree of informational masking as described in the literature [2,4,10,12,15,17,22], but could suggest that cortical sensory gating networks are ‘primed’ for suppressing noise when it contains meaningful content and/or possesses greater similarity with the target signal. While this is pure speculation, it may be relevant for future research to determine how various levels of informational masking in noise affect the gating response, using a wider range of noise types, such as continuous noise, interrupted noise, and one- or two-talker babble.

### 4.3. Study Limitations

This was a preliminary study designed to observe general effects of noise on pre-attentive inhibition via auditory gating, and as such, it enrolled a limited number of participants (n = 13). Although the number of participants is commensurate with several studies referenced in this paper [1,4,9,11,22,59,65,69], future studies should increase participant recruitment and observe effects of background noise on a range of P50 gating responses. Furthermore, behavioral measurements of SPiN were not obtained in this study. Because previous research in our lab suggests that reduced inhibition relates to SPiN deficits in informational masking [27], future studies on the effects of background noise on inhibitory function should include the screening of SPiN performance, as well as regression analyses to examine the relationship between noise effects on gating and behavioral performance. Along these lines, we would like to reiterate the description of the enrolled participants, such that none reported a history of hearing, speech, or language disorders. All participants showed normal hearing with present thresholds ≤ 20 dB HL at each octave within the clinical frequency range. Finally, it is possible that the limited number of components identified using ICA may have influenced the observed gating response in both quiet and background noise by decreasing the amplitude. Care should be taken to optimize the number of components chosen for data analyses, especially to maximize the degraded amplitude of the CAEP gating response in background noise. 

## 5. Conclusions

The findings of this study suggest that background noise negatively affects pre-attentive sensory inhibitory function as measured using the CAEP gating response. Within-group gating was absent for all components in both noise conditions. A reduced activation of gating inhibitory networks was also observed using topographic scalp maps. Although no significant differential effects of noise with low versus high informational masking content on the gating response were found, visual trends for a greater decrease in sensory inhibition in noise with less informational masking were present. Future research studies should aim to increase upon the sample size in this study, to improve its power in order to statistically determine such trends, and should especially include the behavioral performance of subjects in these specific conditions. Finally, the SNR effects of these specific noise types on gating should also be examined.

## Figures and Tables

**Table 1:** Mean latencies and standard deviations for P50, N1, and P2 peak components across listening conditions.

	P50		N1		P2	
	S1 (s.d.)	S2	S1	S2	S1	S2
Quiet	58 ms (11 ms)	58 ms (7 ms)	85 ms (10 ms)	83 ms (21 ms)	116 ms (17 ms)	118 ms (14 ms)
Multi-talker babble-modulated noise	57 ms (14 ms)	51 ms (14 ms)	114 ms (24 ms)	125 ms (14 ms)	185 ms (25 ms)	208 ms (19 ms)
Multi-talker babble	81 ms (5 ms)	86 ms (9 ms)	171 ms (16 ms)	188 ms (15 ms)	204 ms (18 ms)	228 ms (18 ms)

## Data Availability

Please contact the corresponding author for data access.
